# Effect of Linseed Supplementation on Total Longissimus Muscle Lipid Composition and Shelf-Life of Beef From Young Maremmana Bulls

**DOI:** 10.3389/fvets.2018.00326

**Published:** 2019-01-07

**Authors:** Giuseppe Conte, Andrea Serra, Laura Casarosa, Francesca Ciucci, Alice Cappucci, Eleonora Bulleri, Leonor Corrales-Retana, Arianna Buccioni, Marcello Mele

**Affiliations:** ^1^Department of Agriculture, Food and Environment, University of Pisa, Pisa, Italy; ^2^Research Center of Nutraceuticals and Food for Health, University of Pisa, Pisa, Italy; ^3^Dipartimento di Scienze delle Produzioni Agro-alimentari e dell'Ambiente, University of Florence, Florence, Italy

**Keywords:** linseed, fatty acids, CLA isomers, shelf-life, meat

## Abstract

Twenty young Maremmana bulls were randomly allotted to two dietary groups during a finishing period (~90 days): C diet (without lipid supplementation), and L diet (with linseed supplementation, 20% concentrate feed). The aim was to investigate the effects of dietary linseed supplementation on the intramuscular fat composition, and the shelf-life of minced beef. The L diet did not alter the dressing rate and daily weight gain, whereas the C18:3n-3 content in the intramuscular fat significantly increased (from 0.85 to 1.78 g/100 g of total fatty acid, +109%), leading to a reduction in a n-6/n-3 ratio below four and an increase in the overall proportion of long chain n-3 fatty acids in beef lipids. These effects were principally related to differences in the distribution of fatty acids between the neutral and polar lipids. The results demonstrated that linseed supplementation modified the lipid composition of beef, without negatively impacting overall productivity, in the period from weaning the bull until its slaughter. However, the total amount of n-3 fatty acids in the beef of young bulls on the L diet, was not sufficient for the aliquot of humans′ n-3 fatty acid requirements. This was mainly related to the low levels of fat in the beef. Comparing beef samples from animals on the L diet, with samples from animals on the C diet, the higher levels of n-3 polyunsaturated fatty acids led to a significant reduction of shelf-life starting after 2 days of storage at 4°C, because of fatty acid and cholesterol oxidation. The L diet group samples showed a higher level of TBARS (+80%) and COPs (+73%), two important parameters to estimate the oxidation level of beef. This suggests that enriching beef with n-3 fatty acids should be coupled with packaging techniques that consider the higher susceptibility to lipid oxidation. In conclusion, dietary linseed supplementation led to a higher proportion of n-3 fatty acids in beef lipids, however, the natural content of antioxidant substances was not able to protect intramuscular fat from oxidation during storage at 4°C.

## Introduction

An adequate dietary intake of n-3 fatty acids (FAs) can protect one against the risks of cardiovascular disease, diabetes, and obesity as well as some types of cancer (1). There is thus interest in increasing the content of n-3 FAs and other potentially bioactive FAs (i.e., conjugated linoleic acid isomers) in the food chain, including in animal-derived food such as beef (2).

Feeding strategies have therefore been applied in beef cattle to promote the muscle tissue deposition of n-3 polyunsaturated FA (PUFA) and conjugated linoleic acid (CLA) isomers and to lower the n-6/n-3 PUFA ratio (3). The inclusion of α-linolenic acid (C18:3 n-3) sources in the diet of beef cattle, such as well-preserved forage (4), pasture (5), linseed (6), or linseed oil (7) has been found to increase the concentration of some of the long chain n-3 PUFA in intramuscular fat.

The length of the grazing period is fundamental to enrich intramuscular fat with n-3 FA, and at least 100 grazing days are necessary to obtain a stable enrichment of n-3 FA in beef (8). Higher levels of n-3 FA enrichment may be achieved by supplementing grazing heifers with linseed oil-enriched concentrates (8).

Modification of the intramuscular FA profile favors a high susceptibility of unsaturated FA to oxidation, especially those FAs with more than two double bonds. Fatty acid oxidation can produce several secondary products that affect meat color, flavor, and texture (9), with a consequent reduction in the quality and shelf-life of meat.

Similar to FAs, cholesterol oxidation can also generate secondary products, such as oxysterols (10). These metabolites are not associated with a specific off-flavor, but are thought to be very dangerous for human health (11). Lipid oxidation can take place either during the first hours after slaughtering or during the storage time at the retailer.

There are several studies on the effects of dietary linseed supplementation on meat FA composition both from beef cattle (12, 13), lambs (14, 15), and poultry (16, 17). However, the effects of n-3 PUFA supplementation on beef lipid oxidation are less investigated. Previous studies, in fact, have mainly reported on the effects on beef color (18, 19) and/or the content of various oxidation products (20).

The aim of the present study was to investigate the effects of dietary supplementation with extruded linseed during the finishing period of young Maremmana bulls, previously maintained at pasture, on the intramuscular fat composition, and oxidative stability of the intramuscular fat during shelf-life.

## Materials and Methods

### Animal Management, Diet and Samplings

A trial was conducted with 20 young Maremmana bulls. Maremmana are an autochthonous cattle breed from Tuscany (central Italy), usually maintained on native pastures and shrubs. The beef is usually obtained from young bulls slaughtered before 24 months of age, and after 90–100 days (usually from September to November) of a finishing period based on cereals and grass hay (21).

Weaning (at 6 months old) took place at the beginning of winter (December). Throughout the winter period, the animals were collectively maintained in a feedlot with a feeding regimen based on grass hay and a mixture of cereal grains. The average weight of the calves at weaning was 180 ± 29 kg (mean ± s.d.). At the beginning of spring, the animals were moved to the pasture until the finishing period. During this period, the animals grazed the same natural pasture, which consisted of 80 grass, 15 legumes (mainly white clover), and 5% other species. Throughout this period, all the animals had grass hay available on the pasture and received 3 kg per head/per day of a control concentrate (C) (see Table 1 for the ingredients and chemical composition of the concentrate).

**Table 1:** Ingredients and chemical composition of the two concentrate feeds adopted in the experiment.

		**Concentrate feed**	
		**C^b^**	**L^c^**
Corn meal	% DM	40	40
Barley meal	”	37	20
Beet pulp	”	7	10
Wheat bran	”	7	7
Soybean meal	”	6	-
Extruded linseed	”	-	20
Mineral suppl.	”	3	3
DM^a^	% as feed	88.0	88.0
Crude protein	% DM	13.7	13.7
Crude lipids	”	2.8	9.7
Neutral detergent fiber	”	19.2	21.2
Starch	”	48.7	40.2
Ashes	”	5.9	6.2
C16:0	g/100 g of total FA	18.8	14.3
C18:0	”	6.7	3.4
C18:1c9	”	23.7	22.4
C18:2n6	”	35.8	7.8
C18:2n3	”	2.7	42.4
Net energy	kcal/kg DM	2010	2096

a *Dry Matter*;

b *Control concentrate*;

c *Linseed concentrate*.

At the beginning of the finishing period, the animals moved to a feedlot and were randomly allotted to two groups (10 animals per group) according to the concentrate feed offered. The animals of the same treatment were divided into two pens (five animals per pen). One group received 5 kg per head/per day of a concentrate feed containing 20% dry matter of extruded linseed (L group, Table 1). The other group received 5 kg per head/per day of the C concentrate (C group). Concentrate feed was individually administered to animals twice a day. Grass hay was fed *ad libitum* and the average group intake was evaluated. The treatment lasted ~90 days. During the finishing period, the animals were individually weighed on a monthly basis. At the end of the trial, the young bulls were transported to a public abattoir, where they were slaughtered according to EU Regulations. After slaughter, the carcasses were immediately weighed to obtain the hot carcass weight, which was used to assess the dressing percentage. The carcasses were kept at 10°C for 24 h, then refrigerated at 4°C until the 15th day after slaughter. The *longissimus thoracis* muscle (LT, from the 8th to the 20th vertebra) was then removed, vacuum packed, and stored at −80°C until analysis.

For each animal, a 150 g portion of LT was removed from the freezer and, while still partially frozen, was finely blended using a homogenizer. The minced muscle was divided into 3 sub-samples, which were shaped into round patties (2 cm thick). Three patties per animal were prepared, one was immediately analyzed (T0), while the other two were wrapped in oxygen-permeable film which was not in contact with the surface of the meat, and analyzed after 2 (T2) and 6 (T6) days of storage at 4°C in the dark. Meat was minced to evaluate the effect of the shelf-life, on a highly-stressed matrix, to be able to estimate whether the meat of the control group showed a different level of oxidation in these conditions.

The following parameters were thus evaluated: FA composition of total, neutral and polar lipids of beef were determined on T0 samples; while carotenoids, vitamin A, and vitamin E content in total lipids of beef samples and oxidation products of FA and cholesterol were determined on T0, T2, and T6 samples.

### Diet Composition

The ingredients and chemical composition of the two concentrate feeds are reported in Table 1. No antioxidants were added to the experimental diet, as the aim was to estimate the lipid stability against oxidation processes in enriched PUFA meat, without antioxidant protection. Forage and concentrate samples were analyzed for moisture, protein, ether extract, and ash according to official AOAC methods (22). Nitrogen was determined using a protein analyzer (model NA2100, CE Instruments, ThermoQuest Italia, Rodano, Italy), and the ether extract was collected using an ANKOM model XT10 extractor (ANKOM Technology, NY, United States). Fiber fractions were analyzed according to Van Soest et al. (23). The net energy content of the experimental concentrates was estimated according to the NRC (24).

### Color Measurement

For the color measurements, samples were placed on a standard white tile. Color readings were taken at four randomly-selected locations on the upper surface of each round patty in order to obtain a representative mean value. The determination was performed for the round patties at T0, T2, and T6. Muscle color was measured in the CIE L^*^a^*^b^*^ space (25) with an area diameter of 8 mm, specular component included, and 0% UV, D65 standard illuminant, observer angle 10°, and zero and white calibration using a Minolta model CM 2006d spectrophotometer (Konica Minolta Holdings, Inc., Osaka, Japan). Lightness (L^*^), redness (a^*^), and yellowness (b^*^) were recorded, and hue angle (H^*^) and chroma (C^*^) indexes were calculated as H^*^ = tan^−1^(b^*^/a^*^) expressed in degrees, and C^*^ = (a^*2^ + b^*2^)^0.5^ (26). Samples were allowed to bloom for 55–60 min before being measured *in situ*.

### Proximate Composition and Fatty Acid Analysis of Beef

Moisture, crude protein, and ash contents were determined according to AOAC 2000 methods 950.46, 976.05, and 920.153, respectively.

Heme iron was determined following the analytical conditions described by Hornsey (27) with some modifications. Briefly, triplicate samples (2 g) of meat were resuspended in 10 mL of 80% acetone solution acidified with 2% HCl and homogenized by ULTRATURRAX (IKA®-Werke GmbH & Co. KG, Staufen, Germany) for 20 s. Samples were incubated at room temperature for 1 h in the dark, and then centrifuged at 3,000 × g for 10 min at 10°C. The surnatant was analyzed by a spectrophotometer at 640 nm using an acid acetone solution as the blank. The iron was quantified with an external calibration curve, using myoglobin as the standard. The non-Heme iron content was evaluated following Ahn et al. (28).

Total lipids (TL) of beef samples were extracted with a chloroform/methanol solution (2:1, v/v), according to Rodriguez-Estrada et al. (29). TL were then separated into neutral (NL) and polar (PL) lipids by solid phase extraction using silica gel cartridges (LiChrolut Si, 40–63 lm, 500 mg/ml, Standard, Merck KGaA, Darmstadt, Germany), according to Juaneda and Rocquelin (30).

For each lipid fraction (i.e., TL, PL, and NL), FA methyl esters (FAME) were prepared using a methanolic hydrochloric acid solution (10%) according to Christie (31) with some modifications. Briefly, 1 mg of C19:0 as an internal standard was added to 20 mg of lipids and 2 mL of methanolic hydrochloric acid solution (10%). Samples were incubated at 50°C and subsequently FAMEs were extracted with 2 mL of hexane and 2 mL of water. After centrifugation at 3,000 × g per 5 min, the upper phase was transferred into a fresh tube, while the lower phase was washed twice with 2 mL of hexane. FAME were separated and identified using a GC-FID (GC 2000 plus, Shimadzu, Columbia, MD, United States) according to Mele et al. (15).

CLA isomers of TL were separated and quantified by three silver ion HPLC columns (ChromSpher 5 Lipids, Varian, Middelburg, Netherlands; 250 mm 4.6 mm i.d.) according to Sehat et al. (32). Briefly, CLA isomers were eluted using a fresh mixture of acetonitrile 0.1% (v/v) in hexane at a flow of 1 mL/min. The injection loop was 20 μL, and UV detection was performed at a wavelength of 233 nm. Quantitative measurements were performed through a calibration curve, using a high purity individual c9,t11 and t10,c12 CLAs (Matreya Inc., Pleasant Gap PA, United States). The CLA mix standard (Sigma Chemical Co., St. Louis, MO, United States), and published isomeric profiles (33) were also used to help identify the CLA isomers in meat TL. Total FAs of the TL and of the PL and NL fractions were expressed as mg/g fresh meat, whereas individual FAs were expressed as g/100 g of the total FAs of TL, PL or NL; CLA content of TL was expressed as mg/100 g of the total FAs.

### Unsaponifiable Fraction Analysis

The unsaponifiable lipid fraction was obtained according to Sanders et al. (34). Briefly, 300 mg of TL were cold saponified by adding 4.5 mL of ethanolic KOH (4.8% w/v) solution and incubated at room temperature overnight. The unsaponifiable matter was isolated by two washes with 4.5 mL of water and 9 mL of hexane. The nonpolar phase (upper phase) was transferred into a fresh tube and dried by nitrogen gas. Finally, the samples were suspended with 1 mL of methanol. Before the saponification, 100 μL of a solution of dihydrocholesterol in chloroform (2 mg/mL) and 25 μL of a solution of 19-hydroxycholesterol (1 mg/mL) in n-hexane/isopropanol (4/1) as internal standards for cholesterol and cholesterol oxidation products (COPs) analysis, respectively, were added to the TL.

### Analysis for Lipid Oxidation

The lipid oxidation was evaluated by considering the content of free fatty acids (FFA), as an indicator of the lipid hydrolysis, and as an indicator of primary products of lipid oxidation. The secondary oxidation products were evaluated by thiobarbituric acid-reacting substances (TBARS) (as an indicator of polyunsaturated fatty acid oxidation) and COPs analyses.

Free fatty acids were separated from the TL by aminopropyl (NH2) solid-phase extraction (SPE) cartridges (500 mg/ mL) (Varian, Palo Alto, CA, United States) after the addition of the internal standard (free C19:0). Free fatty acids were eluted with a mixture of diethyl ether:acid acetic (98:1, v/v) according to Serra et al. (35). FFAs were methylated using commercial trimethyl-silyl-diazomethane (TMSCHN2) (31) and were separated and quantified under the same conditions used to analyze the total FAME, except for the injection mode which was set as splitless during the first 2 min of the GC run.

The secondary oxidation products of FA were evaluated by the TBARS test, extracting malonyldialdehyde (MDA) with a 5% solution of trichloroacetic acid in water. Samples (1 g) were mixed with a 40 mM solution of thiobarbituric acid (TBA) in water and heated at 93°C for 20 min. MDA content was determined by a spectrophotometer (Cary 50, Varian, Palo Alto, CA, United States) at a wavelength of 532 nm. TBARS were quantified by comparing the absorbance with a calibration curve obtained using a solution of tetraethoxypropane (36).

One-tenth of the unsaponifiable matter was used to determine the total cholesterol, and the remaining part was purified by an NH2-SPE cartridge for COP purification according to Serra et al. (37). Cholesterol and COP fractions were then silylated as described by Serra et al. (37), dried under a nitrogen stream, and dissolved in 300 μL of n-hexane. Both cholesterol and COPs were identified and quantified using a GC-FID (GC 2000 plus, Shimadzu, Columbia, MD, United States) equipped with a VF 1-ms apolar capillary column (25 m × 0.25 mm i.d., 0.25 μm film thickness; Varian, Palo Alto, CA, United States). For cholesterol and COP determination, 2 μL of the sample in hexane was injected into the column with the carrier gas (hydrogen) flux at 1 mL/min, and the split ratio was 1:10. The run was carried out in a constant pressure mode. The oven temperature was held at 250°C for 1 min, increased to 260°C over 20 min at the rate of 0.5°C/min, and then increased to 325°C over 13 min at the rate of 5°C/min, and kept at 325°C for 15 min. Chromatograms were processed with a LabSolution (Shimadzu, Columbia, MD, USA). Cholesterol and COPs were expressed as mg/100 g of muscle.

### Carotenoids, Vitamins A, and E

Muscle concentration of vitamins and carotenoids was determined according to Serra et al. (37). Twenty microliters of each sample were analyzed using a Prostar HPLC (Varian) equipped with a UV-DAD coupled with a fluorescence detector spectra system (model FL3000, ThermoFinnigan, Whaltam, USA) and a C18 reverse phase column (ChromSep HPLC Columns SS 250 × 4.6 mm including Holder with ChromSep guard column Omnisphere 5 C18).

Two solutions were used as the mobile phase: one (A solution) composed of methanol:acetonitrile:water (10:70:20), the other (B solution) composed of methanol:ethyl acetate (70:30). The samples were injected into the column with 90% of solution A and 10% of solution B, maintaining a flow of 1 mL/min for 15 min. Then, a 50:50 ratio between solutions A and B at 1 mL/min flow was kept for 5 min. Finally, the flow rate was increased to 1.5 mL/min with 100% of solution B and maintained for 10 min before returning to the starting conditions. Carotenoids were detected by a UV-DAD detector at 450 nm, vitamin A was detected at 325 nm, while vitamin E was detected by the fluorimeter (excitation λ = 298 nm; emission λ = 340 nm). Quantification was obtained by an external calibration curve, obtained from the retinol, carotenoid and tocopherol standards at concentrations ranging from 0.045 μg/mL to 7 mg/mL. Carotenoids and vitamins were expressed as μg/kg of meat.

### Statistical Analysis

Data on the growth performance, and proximate and FA composition of meat were analyzed using the following linear model, using JMP software (SAS Institute Inc., Cary, NC, United States):

$$\begin{array}{l} y_{\text{ijz}}=\mu +D_{\text{i}}+P_{\text{z}}+\varepsilon_{\text{ijz}} \end{array}$$

where y_ijz_ = dependent variables; Di = fixed effect of the i_th_ diet treatment (control; linseed); P_z_ = random effect of the z_th_ pen (2 levels); ε_ijz_ = random residual.

Data of color and lipid oxidation parameters were analyzed using the following mixed linear model:

$$\begin{array}{l} y_{\text{ijzk}}=\mu +D_{\text{i}}+S_{\text{j}}+D_{\text{i}}xS_{\text{j}}+I_{\text{z}}\left(D\right)+\varepsilon_{\text{ijzk}} \end{array}$$

where y_ijzk_ = dependent variables, D_i_ = fixed effect of the i_th_ diet treatment (control; linseed); S_j_ = fixed effect of the j_th_ storage time (T0, T2, T6); I_z_ = random effect of the z_th_ animal nested within dietary treatment (20 animals); ε_ijzk_ = random residual. Least-square means with their standard errors were reported, and treatment effects were declared significant at *P* < 0.05.

## Results and Discussion

### Growth Performance and Carcass Composition

The two groups of animals showed similar weights at weaning and at the beginning of the finishing period (Table 1).

The average daily gain of the young bulls for the finishing period did not differ across groups (Table 2). This was due to the similar nutritional characteristics of the diets (Table 1). The average daily gain values were consistent with previous studies, based on the feedlot system during the finishing period of young Maremmana bulls (21).

**Table 2:** Effect of linseed supplementation on growth performance and meat composition.

		**Diet treatment**		**SEM^a^**	***P*-value**
		**C^b^**	**L^c^**		
Weaning weight	kg	181.90	177.30	9.30	0.73
Live weight at initiation of trial^d^	“	471.70	437.20	20.44	0.25
Live weight at the end of the trial^e^	“	566.70	532.20	20.44	0.25
Average daily weight gain^f^	“	1.05	1.04	0.10	0.20
Hot carcass weight	“	243.30	238.40	8.40	0.68
Hot right half carcass	“	123.40	121.40	4.10	0.74
Cold right half carcass	“	118.30	115.20	4.00	0.60
Dressing percentage	%	52.90	55.00	0.90	0.12
Carcass weight loss	kg	5.00	6.10	1.30	0.57
**Longissimus muscle composition**					
Moisture	g/100 g meat	75.66	74.90	0.30	0.10
Protein	“	22.01	21.89	0.32	0.12
Ash	“	1.11	1.10	0.01	0.71
Fat	“	1.53	1.72	0.14	0.09
Total Iron	mg/100 g meat	0.98	1.03	0.05	0.49
no-Heme Iron	“	0.18	0.18	0.01	0.58
Heme Iron	“	0.80	0.86	0.05	0.39
Organic Iron	%	83.30	83.50	1.12	0.99

a *Standard Error of Mean*.

b *Control group*.

c *Linseed group*.

d *Weight of bulls at beginning of finishing period*.

e *Weight of bulls before the slaughter*.

f *The average daily weight refers to the trial period, during the finishing*.

The inclusion of linseed in the finishing diet did not affect the growth performance (Table 2), confirming findings previously reported both for young bulls (18) and lambs (14, 15). It is important to consider that when treatments are applied during the finishing phase, differences due to energy supplementation were observed in terms of fat deposition, which could reduce treatment differences in average daily weight gain. The lack of a treatment effect on weight gain in the L diet compared to the C diet observed in this study are consistent with the results previously obtained in grazing steers supplemented with increasing levels of linseed (38). Generally, dressing percentage and the other growth performance parameters depend mainly on breed, slaughter weight, age, and sex, (38). Since the bulls were all of the same breed, age, and sex, it is plausible that there were no differences in growth performance.

The value of dressing percentage (Table 2) observed in this study is consistent with the data previously reported in the literature of other rustic breeds (39).

### Proximate and Fatty Acid Composition of Beef

Moisture, protein, ash, lipids, and iron contents of beef samples are reported in Table 2. The inclusion of linseed in the diet did not modify any characteristics of the proximate composition, probably due to the comparable muscle development throughout the experimental period. The intramuscular fat content did not differ, as expected in animals with a similar average daily gain and in animals slaughtered at the same age and weight. Overall, beef samples were very lean, the content of intramuscular fat being lower than 2%, irrespective of the dietary treatment (Table 2). Very lean meat is a typical characteristic of beef from Maremmana cattle and other autochthonous breeds from Italy (21, 34).

The intramuscular FA content and the composition of the TL, NL, and PL are presented in Tables 3–**5**, respectively. The linseed supplementation did not significantly affect the total FA concentration of neither the TL nor the NL and PL fractions, which averaged 11.7 ± 1.0, 8.6 ± 1.0, and 3.0 ± 0.4 mg/g muscle, respectively. The average NL:PL ratio did not change across treatments and was 3.2 ± 0.5 (on average, 27% of PL and 74% of NL).

**Table 3:** Effect of dietary linseed supplementation on total fatty acid concentration (mg/g fresh muscle) and composition (g/100 g total fatty acid) of total lipids from the longissimus muscle.

**Fatty acids**	**Diet treatments**		**% change^a^**	**SEM^b^**	***P*-value**
	**C^c^**	**L^d^**			
Total FA	12.24	11.10		1.24	0.52
C10:0	0.19	0.18		0.01	0.65
C12:0	0.08	0.08		0.01	0.33
C14:0 iso	0.09	0.10	+11	0.01	< 0.01
C14:0	2.82	3.08		0.16	0.26
C15:0 iso	0.19	0.14	−26	0.01	< 0.01
C14:1 c9	0.52	0.71		0.07	0.08
C15:0 anteiso	0.22	0.16	−27	0.01	< 0.01
C15:0	0.44	0.33	−25	0.02	< 0.01
C16:0 iso	0.24	0.17	−29	0.01	< 0.01
C16:0	19.57	19.29		0.46	0.68
C16:1 t9	0.12	0.16	+33	0.01	0.02
C16:1 c7	0.21	0.21		0.01	0.36
C16:1 c9	1.84	2.17		0.13	0.09
C17:0 iso	0.52	0.46	−12	0.01	< 0.01
C17:0 anteiso	0.65	0.54	−17	0.02	< 0.01
C17:0	1.04	0.85	−18	0.04	< 0.01
C17:1 c9	0.55	0.51		0.02	0.18
C18:0 iso	0.17	0.13	−23	0.01	< 0.01
C18:0	17.58	15.98		0.85	0.20
C18:1 t4	0.02	0.02		0.01	0.51
C18:1 t5	0.02	0.02		0.01	0.46
C18:1 t6–8	0.33	0.29		0.03	0.39
C18:1 t9	0.34	0.36		0.01	0.19
C18:1 t10	0.47	0.89	+89	0.12	0.03
C18:1 t11	2.06	2.66		0.23	0.09
C18:1 t12	0.28	0.66		0.14	0.07
C18:1 c9	32.51	32.51		1.01	0.99
C18:1 c11	1.59	1.84	−16	0.05	< 0.01
C18:1 c12	0.28	0.75	+168	0.02	< 0.01
C18:2 t9t12	0.25	0.35		0.04	0.10
C18:2 t11c15	0.10	0.95	+850	0.03	< 0.01
C18:2 n-6	8.24	7.25		0.65	0.29
C18:3 n-3	0.85	1.78	+109	0.07	< 0.01
C20:0	0.14	0.12	−14	0.01	0.02
C20:1 c11	0.18	0.19		0.02	0.60
C18:3 c9t11c15	0.07	0.07		0.01	0.82
C20:2 c11c14	0.08	0.07		0.01	0.40
C20:3 n-6	0.79	0.43	−45	0.07	< 0.01
C20:4 n-6	2.51	1.61	−35	0.26	0.02
C20:5 n-3	0.35	0.44		0.05	0.23
C22:4 n-6	0.24	0.09	−63	0.02	< 0.01
C22:5 n-3	0.75	0.71		0.09	0.79
C22:6 n-3	0.08	0.08		0.01	0.09
Total CLA	0.52	0.76	+46	0.06	< 0.01
SFA	44.06	41.66		1.29	0.20
PUFA	14.88	14.62		1.16	0.88
MUFA	41.06	43.71		1.23	0.14
PUFA n-6	12.72	11.23		1.01	0.31
PUFA n-3	2.21	4.05	+83	0.20	< 0.01
MCFA	29.10	28.90		0.67	0.84
LCFA	70.71	70.91		0.66	0.83
BCFA	2.21	1.75	−21	0.06	< 0.01
BCFAiso	1.34	1.05	−22	0.03	< 0.01
BCFAanteiso	0.87	0.71	−18	0.03	< 0.01
OCFA	2.03	1.69	−17	0.06	< 0.01
TFA	3.90	5.41	+39	0.20	< 0.01
n-6/n-3	9.94	4.66	−53	0.44	< 0.01

a *percentage of change after linseed supplementation*.

b *Standard Error of Mean*.

c *Control group*.

d *Linseed group. SFA, Saturated Fatty Acids (C10:0 + C12:0 + C14:0 iso + C14:0 + C15:0 iso + C15:0 anteiso + C15:0 + C16:0 iso + C16:0 + C17:0 iso + C17:0 anteiso + C17:0 + C18:0 iso + C18:0 + C20:0); PUFA, Polyunsaturated Fatty Acids (C18:2 t9t12 + C18:2 t11c15 + C18:2 n-6 + C18:3 n-3 + C18:3 c9t11c15 + C20:2c11c14 + C20-3 n-6 + C20:4 n-6 + C20:5 n-3 + C22:4 n-6 + C22:5 n-3 + C22:6 n-3 + total CLA); MUFA, Monounsaturated Fatty Acids (C14:1 c9 + C16:1 t9 + C16:1 c7 + C16:1 c9 + C17:1 c9 + C18:1 t4 + C18:1 t5 + C18:1 t6-8 + C18:1 t9 + C18:1 t10 + C18:1 t11 + C18:1 t12 + C18:1 c9 + C18:1 c11 + C18:1 c12 + C20:1 c11); MCFA, Medium Chain Fatty Acids (C10:0 + C12:0 + C14:0 iso + C14:0 + C15:0 iso + C14:1 c9 + C15:0 anteiso + C15:0 + C16:0 iso + C16:0 + C16:1 t9 + C16:1 c7 + C16:1 c9 + C17:0 iso + C17:0 anteiso + C17:0 + C17:1 c9); LCFA, Long Chain Fatty Acids (C18:0iso + C18:0 + C18:1 t4 + C18:1 t5 + C18:1 t6-8 + C18:1 t9 + C18:1 t10 + C18:1 t11 + C18:1 t12 + C18:1 c9 + C18:1 c11 + C18:1 c12 + C18:2 t9t12 + C18:2 t11c15 + C18:2 n-6 + C18:3 n-3 + C20:0 + C18:3 c9t11c15 + C20:2c11c14 + C20-3 n-6 + C20:4 n-6 + C20:5 n-3 + C22:4 n-6 + C22:5 n-3 + C22:6 n-3 + total CLA); BCFA, Branched Chain Fatty Acids (C14:0 iso + C15:0 iso +C15:0 anteiso + C16:0 iso + C17:0 iso + C17:0 anteiso + C18:0 iso); OCFA, Odd Chain Fatty Acids (C15:0 iso + C15:0 anteiso + C15:0 + C17:0 iso + C17:0 anteiso + C17:0 + C17:1 c9); TFA, Trans Fatty Acids (C16:1 t9 +C18:1 t4 + C18:1 t5 + C18:1 t6-t8 + C18:1 t9 + C18:1 t10 + C18:1 t11 + C18:1 t12+ C18:2 t9t12); total CLA, sum of conjugated linoleic acid isomers*.

The total FA in muscle reflects the weighted combination of PL and NL fractions. Overall, TL were characterized by 43% saturated FA (SFA), 42% monounsaturated FA (MUFA), and 15% PUFA. These data are similar to those previously reported in other studies (40, 41).

The content of SFA and MUFA in the TL was not significantly affected by linseed supplementation. Previous studies reported that SFA decreased and MUFA increased when linseed was included in the diet of beef cattle (18). It is likely that our data showed no differences because beef samples had a lower overall fat content. Since SFA and MUFA are more selectively accumulated in the fat depots, the effect of linseed supplementation for these FA classes was probably less evident. The PUFA content in the TL was consistent with data previously reported for beef samples from young bulls with a similar content of TL in the muscle (14–18%) (41). For both experimental groups, the major FA in TL were C18:1c9 (32% TL), C16:0 (19% TL), and C18:0 (16% TL). Their content in the TL was not affected by the treatments (Table 3). The linseed in the diet significantly increased the level of C18:3n-3 (+ 109%) in the TL. However, the content of C18:3n-3 in the TL of samples from C diet animals was higher than that observed in beef of animals fed a diet that was not supplemented with linseed (0.85 vs. < 0.5%), as revealed in previous studies (18, 39, 42, 43). There are two possible reasons for these findings, firstly, the beef samples collected in the present study were very lean, thus the polar lipids were more representative, including C18:3n-3 which accumulates preferentially in the cell membranes (14). In addition, animals from both groups grazed in the period prior to the finishing phase, thus the level of C18:3n-3 may have increased. Finally, some differences may also originate from the different ways of calculating the relative proportion of FAs. In the present study, the proportion of a given FA was determined by dividing the area of a single FA by the total area detected, whereas in some studies, the proportion of a given FA was obtained by dividing the area of a single FA by the area of all the identified FAs.

The higher level of C18:3n-3 (*P* < 0.01) in the L diet was associated with a significant increase in the intermediate products of ruminal biohydrogenation of C18:3n-3: C18:2t11,c15 (+899%, *P* < 0.01), C18:1c11 (+15%, *P* < 0.01), and C18:1c12 (+167%, *P* < 0.01). Although C18:2 c9t11 was not intermediates of C18:3 n-3 biohydrogenation, the content of this FA significantly increased with linseed supplementation (+45%, *P* < 0.04). The low conversion of octadecenoates to C18:0 probably led to an accumulation of C18:1 t11 isomers in the rumen. After intestinal absorption, C18:1 t11 was converted into C18:2 c9t11 by Δ9-desaturase (7), thus contributing to the increase in this FA in the beef samples from animals that were fed the L diet.

At the same time, a significant reduction was observed in the branched-chain FA (BCFA, *P* < 0.01) and odd chain FA (OCFA, *P* < 0.01) in the TL from the L diet samples: −21 and −17% for BCFA and OCFA, respectively. Since BCFA and OCFA were mainly derived from rumen microbial biomass, the supplementation of unprotected vegetable oil with extruded linseed could have negatively affected the rumen microbial metabolism and ecology by lowering the microbial biomass flow to the duodenum (7). Total lipids from the L diet group also showed a significant lower level of omega-6 LCFA: C20:3n-6 (−45%, *P* < 0.01), C20:4n-6 (−36%, *P* = 0.02) and C22:4n-6 (−62%, *P* < 0.01). Data found in the present work agrees with the FA profile found in young bulls in other studies (42, 43).

Overall, the FA compositions of PL and NL were both influenced by linseed supplementation, as previously reported by Scollan et al. (3). The FA composition of the NL fraction was characterized by a high proportion of SFA and MUFA, whereas the PL fraction showed a high proportion of PUFAs as reported in a previous work (14) (**Table 5**).

Similar to findings for the TL, the highest FAs in the NL were C18:1c9 (36% of NL), C16:0 (21% of NL), and C18:0 (18% of NL) (Table 4). The contents of BCFA (−21%, *P* < 0.01), C15:0 (−27%, *P* < 0.01), C17:0 (−17%, *P* < 0.01), C18:2n-6 (−18%, *P* < 0.01), C20:0 (−29%, *P* = 0.03), C20:3n-6 (−52%, *P* < 0.01), and C22:4n-6 (−59%, *P* < 0.01) were lower in the L diet group samples. Conversely, the linseed diet was associated with a higher content of C18:1t10 (+102%, *P* < 0.01), C18:1t11 (+45%, *P* < 0.01), C18:1c11 (+20%, *P* = 0.02), C18:1c12 (+123%, *P* < 0.01), C18:2t11,c15 (+842%, *P* < 0.01), C18:3n-3 (+63%, *P* = 0.02), C18:3c9,t11,c15 (+335%, *P* < 0.01).

**Table 4:** Effect of dietary linseed supplementation on total fatty acid concentration (mg/g fresh muscle) and composition (g/100 g total fatty acid) of neutral lipids from the longissimus muscle.

**Fatty acids**	**Diet treatment**		**SEM^a^**	***P*-value**
	**C^b^**	**L^c^**		
Total FA	8.86	8.48	1.00	0.79
C10:0	0.19	0.19	0.01	0.94
C12:0	0.12	0.12	0.01	0.83
C14:0 iso	0.12	0.07	0.01	< 0.01
C14:0	3.63	3.77	0.13	0.45
C15:0 iso	0.25	0.18	0.01	< 0.01
C14:1 c9	0.69	0.80	0.07	0.30
C15:0 anteiso	0.28	0.20	0.01	< 0.01
C15:0	0.53	0.39	0.02	< 0.01
C16:0 iso	0.28	0.19	0.01	< 0.01
C16:0	21.59	20.90	0.35	0.18
C16:1 t9	0.05	0.05	0.01	0.58
C16:1 c7	0.22	0.23	0.01	0.77
C16:1 c9	2.27	2.48	0.15	0.30
C17:0 iso	0.52	0.42	0.02	< 0.01
C17:0 anteiso	0.77	0.62	0.02	< 0.01
C17:0	1.19	0.99	0.04	< 0.01
C17:1 c9	0.59	0.54	0.02	0.10
C18:0 iso	0.07	0.08	0.02	0.64
C18:0	19.11	17.57	0.74	0.16
C18:1 t4	0.03	0.02	0.00	0.09
C18:1 t5	0.02	0.02	0.00	0.86
C18:1 t6–8	0.38	0.43	0.03	0.30
C18:1 t9	0.43	0.43	0.02	0.72
C18:1 t10	0.58	1.18	0.12	< 0.01
C18:1 t11	2.22	3.22	0.22	< 0.01
C18:1 t12	0.48	0.34	0.21	0.64
C18:1 c9	36.33	35.55	0.67	0.42
C18:1 c11	1.35	1.62	0.07	0.02
C18:1 c12	0.28	0.63	0.02	< 0.01
C18:2 t9t12	0.24	0.37	0.05	0.09
C18:2 t11c15	0.12	1.12	0.04	< 0.01
C18:2 n-6	2.51	2.06	0.10	< 0.01
C18:3 n-3	0.53	0.86	0.09	0.02
C20:0	0.18	0.13	0.01	0.03
C20:1 c11	0.19	0.17	0.03	0.75
C18:3 c9t11c15	0.02	0.07	0.01	< 0.01
C20:2 c11c14	0.02	0.02	0.00	0.74
C20:3 n-6	0.09	0.04	0.01	< 0.01
C20:4 n-6	0.14	0.09	0.03	0.23
C20:5 n-3	0.04	0.02	0.01	0.47
C22:4 n-6	0.03	0.01	0.00	< 0.01
C22:5 n-3	0.10	0.07	0.01	0.05
C22:6 n-3	0.01	0.01	0.00	0.14
Total CLA	0.66	0.87	0.05	0.01
SFA	48.92	46.13	0.75	0.02
PUFA	4.51	5.29	0.34	0.13
MUFA	46.54	48.39	0.74	0.09
PUFA n6	3.79	3.40	0.30	0.38
PUFA n3	0.83	2.27	0.07	< 0.01
MCFA	33.18	31.94	0.51	0.10
LCFA	66.60	67.86	0.51	0.10
BCFA	2.28	1.79	0.10	< 0.01
BCFAiso	1.24	1.00	0.05	< 0.01
BCFAanteiso	1.04	0.79	0.07	0.02
OCFA	2.35	2.16	0.18	0.43
TFA	4.82	6.78	0.32	< 0.01
n6/n3	1.47	1.65	0.05	0.02

a *Standard Error of Mean*.

b *Control group*.

c *Linseed group. SFA, Saturated Fatty Acids (C10:0 + C12:0 + C14:0 iso + C14:0 + C15:0 iso + C15:0 anteiso + C15:0 + C16:0 iso + C16:0 + C17:0 iso + C17:0 anteiso + C17:0 + C18:0 iso + C18:0 + C20:0); PUFA, Polyunsaturated Fatty Acids (C18:2 t9t12 + C18:2 t11c15 + C18:2 n-6 + C18:3 n-3 + C18:3 c9t11c15 + C20:2c11c14 + C20-3 n-6 + C20:4 n-6 + C20:5 n-3 + C22:4 n-6 + C22:5 n-3 + C22:6 n-3 + total CLA); MUFA, Monounsaturated Fatty Acids (C14:1 c9 + C16:1 t9 + C16:1 c7 + C16:1 c9 + C17:1 c9 + C18:1 t4 + C18:1 t5 + C18:1 t6-8 + C18:1 t9 + C18:1 t10 + C18:1 t11 + C18:1 t12 + C18:1 c9 + C18:1 c11 + C18:1 c12 + C20:1 c11); MCFA, Medium Chain Fatty Acids (C10:0 + C12:0 + C14:0 iso + C14:0 + C15:0 iso + C14:1 c9 + C15:0 anteiso + C15:0 + C16:0 iso + C16:0 + C16:1 t9 + C16:1 c7 + C16:1 c9 + C17:0 iso + C17:0 anteiso + C17:0 + C17:1 c9); LCFA, Long Chain Fatty Acids C18:0iso + C18:0 + C18:1 t4 + C18:1 t5 + C18:1 t6-8 + C18:1 t9 + C18:1 t10 + C18:1 t11 + C18:1 t12 + C18:1 c9 + C18:1 c11 + C18:1 c12 + C18:2 t9t12 + C18:2 t11c15 + C18:2 n-6 + C18:3 n-3 + C20:0 + C18:3 c9t11c15 + C20:2c11c14 + C20-3 n-6 + C20:4 n-6 + C20:5 n-3 + C22:4 n-6 + C22:5 n-3 + C22:6 n-3 + total CLA); BCFA, Branched Chain Fatty Acids (C14:0 iso + C15:0 iso +C15:0 anteiso + C16:0 iso + C17:0 iso + C17:0 anteiso + C18:0 iso); OCFA, Odd Chain Fatty Acids (C15:0 iso + C15:0 anteiso + C15:0 + C17:0 iso + C17:0 anteiso + C17:0 + C17:1 c9); TFA, Trans Fatty Acids (C16:1 t9 +C18:1 t4 + C18:1 t5 + C18:1 t6-t8 + C18:1 t9 + C18:1 t10 + C18:1 t11 + C18:1 t12+ C18:2 t9t12); total CLA, sum of conjugated linoleic acid isomers*.

The most concentrated FAs in the PL were C18:2n-6 (24%), C18:1c9 (16%), C16:0 (15% of PL), and C18:0 (13% of PL). Their content in PL did not show significant differences between the two groups. On the other hand, the content of long chain PUFA n-6 was significantly lower in the PL in the L diet group. Specifically, C20:4n-6 decreased (*P* < 0.01) from 8% in the animals fed the control diet to 6% in the young bulls fed the L diet (Table 5). Linseed supplementation did not affect the SFA, except for C17:0, which decreased significantly (−14%, *P* = 0.02). Monounsaturated FAs were also unaffected by the linseed supplementation as revealed by Corazzin et al. (44), apart from C18:1 trans isomers which increased significantly, confirming findings reported in the literature (45–47).

**Table 5:** Effect of dietary linseed supplementation on total fatty acid concentration (mg/g fresh muscle) and composition (g/100 g total fatty acid) of polar lipids from the longissimus muscle.

**Fatty acids**	**Diet treatment**		**SEM^a^**	***P*-value**
	**C^b^**	**L^c^**		
Total Fatty Acids	3.38	2.62	0.42	0.22
C10:0	0.27	0.33	0.04	0.30
C12:0	0.07	0.09	0.02	0.42
C14:0 iso	0.00	0.00	0.00	0.33
C14:0	0.66	0.61	0.15	0.82
C15:0 iso	0.05	0.03	0.01	0.13
C14:1 c9	0.09	0.08	0.03	0.94
C15:0 anteiso	0.07	0.05	0.01	0.12
C15:0	0.24	0.21	0.01	0.19
C16:0 iso	0.14	0.11	0.01	0.04
C16:0	15.15	15.97	0.36	0.13
C16:1 t9	0.36	0.60	0.05	< 0.01
C16:1 c7	0.16	0.15	0.01	0.64
C16:1 c9	0.68	0.58	0.09	0.58
C17:0 iso	0.55	0.56	0.05	0.88
C17:0 anteiso	0.27	0.27	0.02	0.84
C17:0	0.62	0.53	0.02	0.02
C17:1 c9	0.42	0.33	0.02	< 0.01
C18:0 iso	0.10	0.06	0.01	< 0.01
C18:0	12.76	12.91	0.34	0.76
C18:1 t6–8	0.10	0.11	0.01	0.84
C18:1 t9	0.25	0.19	0.08	0.61
C18:1 t10	0.25	0.46	0.07	0.05
C18:1 t11	0.72	1.04	0.10	0.04
C18:1 t12	0.20	0.27	0.02	0.02
C18:1 c9	17.43	15.21	1.15	0.19
C18:1 c11	1.85	1.92	0.07	0.48
C18:1 c12	0.42	1.27	0.06	< 0.01
C18:2 t9t12	0.14	0.16	0.02	0.30
C18:2 t11c15	0.17	0.27	0.03	0.03
C18:2 n-6	24.37	25.15	1.13	0.63
C18:3 n-3	1.75	4.06	0.09	< 0.01
C20:0	0.11	0.10	0.01	0.37
C20:1 c11	0.13	0.11	0.02	0.59
C18:3 c9t11c15	0.29	0.20	0.02	0.01
C20:2 c11c14	0.23	0.24	0.01	0.72
C20:3 n-6	2.71	1.87	0.17	< 0.01
C20:4 n-6	8.56	6.57	0.40	< 0.01
C20:5 n-3	1.25	1.75	0.09	< 0.01
C22:4 n-6	0.75	0.39	0.03	< 0.01
C22:5 n-3	2.43	2.73	0.13	0.12
C22:6 n-3	0.22	0.30	0.03	0.25
Total CLA	0.22	0.25	0.04	0.67
SFA	31.11	33.24	1.06	0.17
PUFA	43.41	44.19	1.84	0.77
MUFA	25.48	23.86	1.66	0.50
PUFA n6	37.62	36.16	1.69	0.55
PUFA n3	7.33	9.48	0.83	0.09
MCFA	19.58	20.29	0.90	0.41
LCFA (>17)	80.14	79.36	0.58	0.36
BCFA	1.13	2.22	0.91	0.41
BCFA iso	0.79	0.64	0.08	0.21
BCFA anteiso	0.35	1.58	0.91	0.35
OCFA	1.28	1.09	0.05	0.01
TFA	3.36	3.07	0.86	0.82
n6/n3	15.94	10.90	0.95	< 0.01

a *Standard Error of Mean*.

b *Control group*.

c *Linseed group. SFASaturated Fatty Acids (C10:0 + C12:0 + C14:0 iso + C14:0 + C15:0 iso + C15:0 anteiso + C15:0 + C16:0 iso + C16:0 + C17:0 iso + C17:0 anteiso + C17:0 + C18:0 iso + C18:0 + C20:0); PUFA, Polyunsaturated Fatty Acids (C18:2 t9t12 + C18:2 t11c15 + C18:2 n-6 + C18:3 n-3 + C18:3 c9t11c15 + C20:2c11c14 + C20-3 n-6 + C20:4 n-6 + C20:5 n-3 + C22:4 n-6 + C22:5 n-3 + C22:6 n-3 + total CLA); MUFA, Monounsaturated Fatty Acids (C14:1 c9 + C16:1 t9 + C16:1 c7 + C16:1 c9 + C17:1 c9 + C18:1 t4 + C18:1 t5 + C18:1 t6-8 + C18:1 t9 + C18:1 t10 + C18:1 t11 + C18:1 t12 + C18:1 c9 + C18:1 c11 + C18:1 c12 + C20:1 c11); MCFA, Medium Chain Fatty Acids (C10:0 + C12:0 + C14:0 iso + C14:0 + C15:0 iso + C14:1 c9 + C15:0 anteiso + C15:0 + C16:0 iso + C16:0 + C16:1 t9 + C16:1 c7 + C16:1 c9 + C17:0 iso + C17:0 anteiso + C17:0 + C17:1 c9); LCFA, Long Chain Fatty Acids C18:0iso + C18:0 + C18:1 t4 + C18:1 t5 + C18:1 t6-8 + C18:1 t9 + C18:1 t10 + C18:1 t11 + C18:1 t12 + C18:1 c9 + C18:1 c11 + C18:1 c12 + C18:2 t9t12 + C18:2 t11c15 + C18:2 n-6 + C18:3 n-3 + C20:0 + C18:3 c9t11c15 + C20:2c11c14 + C20-3 n-6 + C20:4 n-6 + C20:5 n-3 + C22:4 n-6 + C22:5 n-3 + C22:6 n-3 + total CLA); BCFA, Branched Chain Fatty Acids (C14:0 iso + C15:0 iso +C15:0 anteiso + C16:0 iso + C17:0 iso + C17:0 anteiso + C18:0 iso); OCFA, Odd Chain Fatty Acids (C15:0 iso + C15:0 anteiso + C15:0 + C17:0 iso + C17:0 anteiso + C17:0 + C17:1 c9); TFA, Trans Fatty Acids (C16:1 t9 +C18:1 t4 + C18:1 t5 + C18:1 t6-t8 + C18:1 t9 + C18:1 t10 + C18:1 t11 + C18:1 t12+ C18:2 t9t12); total CLA, sum of conjugated linoleic acid isomers*.

In addition, samples from the L diet animals showed a significant reduction in all the n-6 very long chain PUFAs (C ≧ 20), which ranged from −23% for C20:4n-6 (*P* < 0.01) to −40% for C22:4 n-6 (*P* < 0.01). On the other hand, the contents of C16:1t9 (*P* < 0.01), C18:1t10 (*P* = 0.05), C18:1t11 (P = 0.04), C18:1t12 (*P* < 0.02), C18:1c12 (*P* < 0.01), C18:2t11c15 (*P* = 0.03), C18:3n-3 (*P* < 0.01), and C18:3c9,t11,c15 (*P* = 0.01) were significantly higher in the PL of the L diet animals.

The most abundant dietary FAs (C18:2 n-6 and C18:3 n-3) clearly had a selective deposition in both NL and PL lipid fractions. In fact, 18:2n-6 and 18:3n-3 were selectively incorporated in the PL, however, the selectivity for the PL was higher for 18:2n-6 (9.7) than for 18:3n-3 (3.3).

This pattern of FA incorporation into the PL fraction was consistent with data reported in previous studies (14, 35), confirming the metabolic control of dietary FA distribution according to the different lipid fractions.

The FA composition of the PL is the principal determinant of membrane fluidity and, consequently cellular metabolism (48). In this work, linseed supplementation was found to increase the proportion of C18:3n-3 (+132%) in intramuscular fat, with a significant increase in the unsaturation level in the PL fraction. The simultaneous reduction in the very long chain (LC) PUFA n-6 in the same lipid fraction, could be a metabolic response in order to maintain the membrane fluidity constant, as suggested by Scislowski et al. (49). This partial substitution of n-6 PUFA by n-3 PUFA in the PL is probably due to competition between C18:2n-6 and C18:3n-3 for desaturation and elongation enzymes, which might affect the conversion to long chain derivatives (50). The higher proportion of n-3 LC-PUFA is likely due to the preference of these enzymes for C18:3n-3 (50).

The n-6/n-3 ratio is highly influenced by linseed supplementation. Generally, this ratio is affected by the FA composition of the diet administered to the animals (44). In the present study linseed supplementation decreased the n-6/n-3 ratio of intramuscular lipids from 9.9 to 4.6 (Table 3), approaching the maximum value recommended for the human diet (51). Similar results were obtained by Albertì et al. (18) in Pirenaica calves (n-6/n-3 = 5.3). Similar or lower values have been reported after feeding cows with diets based on grass (51), or grass silage supplemented with linseed or fish oil (44), or corn silage supplemented with linseed (44).

In this study, the improvement in the n-6/n-3 ratio in the TL was entirely due to the increase in n-3 PUFA, as the n-6 PUFA content did not change, as also reported by Gonzàlez et al. (12). The positive effects of n-3 PUFA on human health are related to the actual dietary intake of n-3 PUFA. We found that 100 g of beef from animals fed the C diet provided nearly 10 mg of C18:3n-3 and 5 mg of n-3 LC-PUFA (EPA + DHA). The same amount of beef from animals on the L diet provided nearly 20 mg of C18:3n-3 and 6 mg of n-3 LC-PUFA. These values accounted for 1–2% of the average recommended daily intake for the human diet both in the case of C18:3n-3 (2,000 mg/person/day) and EPA and DHA (52). Therefore, although linseed supplementation was effective in significantly increasing the n-3 PUFA content of intramuscular fat, Maremmana beef may provide a very small contribution to the overall n-3 PUFA intake in the human diet due to the very lean meat and because of the effect of ruminal biohydrogenation, which reduces the transfer of C18:3 n-3 from the diet to the animal tissues.

The distribution pattern of CLA isomers in the intramuscular fat is reported in Table 6. The predominant isomer in all samples was c9,t11 CLA, representing more than 75% of the total CLA content. Linseed supplementation significantly increased the concentration of c9,t11CLA (+38%), which was preferentially accumulated in the NL fraction rather than in the PL (more than 90% of total CLA was in NL), as reported in previous works (15).

**Table 6:** Effect of dietary linseed supplementation on CLA isomer concentration (mg/100 g Total Lipids).

**CLA isomers**	**Diet treatment**		**SEM^a^**	***P*-value**
	**C^b^**	**L^c^**		
12/14 t/t	1.87	6.97	0.53	< 0.01
11/13 t/t	6.13	16.28	0.78	< 0.01
10/12 t/t	3.43	2.87	0.20	0.06
9/11 t/t	5.76	7.76	0.93	0.16
8/10 t/t	1.51	1.15	0.09	< 0.01
7/9 t/t	2.55	1.92	0.22	0.06
6/8 t/t	1.72	0.88	0.18	< 0.01
11/13 t/c	0.92	10.17	0.85	< 0.01
11/13 c/t	0.19	0.96	0.07	< 0.01
10/12 t/c	9.59	26.27	2.92	< 0.01
9/11 c/t	243.13	335.04	29.78	0.04
8/10 c/t	5.33	7.54	0.61	0.02
7/9 t/c	29.53	36.55	3.12	0.13

a *Standard Error of Mean*;

b *Control group*;

c *Linseed group*.

Linseed supplementation resulted in a higher content of t12,t14CLA (3.7 fold, P < 0.01), t11,t13CLA (2.6 fold, *P* < 0.01), t11,c13CLA (11.0 fold, *P* < 0.01), c11,t13CLA (5.0 fold *P* < 0.01), t10,c12CLA (2.7 fold, *P* < 0.01), c8,t10CLA (1.4 fold, *P* = 0.02) in TL. Diets containing a high level of linolenic acid in the fat, such as fresh forage, pasture, or concentrate with linseed resulted in an increased deposition of c9,t11CLA (53). Data from this study showed that linseed supplementation also induced a higher concentration of t11,c13CLA, c11,t13CLA, and t12,t14CLA in intramuscular fat. Similar results have been obtained in dairy cows fed a high C18:3n-3 diet (51). Linolenic acid, in fact, may be an indirect precursor of t11,c13CLA, c11,t13CLA, and t12,t14CLA, however the specific metabolic pathway is still unknown (53). The content of the second most abundant CLA isomer (t7,c9CLA) was not affected by linseed supplementation, confirming findings reported by Dannenberger et al. (53) in young grass-fed bulls. Since t7,c9CLA is endogenously produced by the Δ9-desaturation of the C18:1t7 isomer (53), linseed supplementation probably did not induce a rumen accumulation of C18:1t7.

### Lipid Oxidation of Beef

#### Color

Data regarding meat color are reported in Table 7. At time 0, minced beef samples showed a high lightness value (49.7 ± 1.43), a low redness value (12.7 ± 1.07) and a relatively low yellowness value (12.5 ± 1.31), irrespectively of the treatment.

**Table 7:** Effect of storage time on color parameters.

	**Control (C)**			**Linseed (L)**			**SEM^a^**	***P*-value**
	**T0**	**T2**	**T6**	**T0**	**T2**	**T6**		
^L*^	49.54^A^	45.07^B^	43.64^B^	49.96^A^	46.86^B^	44.58^B^	1.43	< 0.01
^a*^	13.04^A^	7.43^B^	7.38^B^	12.27^A^	6.75^B^	6.35^B^	1.08	< 0.01
^b*^	12.89^A^	10.14^B^	12.81^A^	12.15^A^	8.57^B^	11.25^A^	1.17	< 0.01
^H*^	44.15^C^	53.24^B^	61.19^A^	44.77^C^	51.80^B^	61.10^A^	1.87	< 0.01
^C*^	18.39^A^	12.61^B^	14.87^B^	17.29^A^	10.95^B^	13.08^B^	1.37	< 0.01

a *Standard Error of Mean; Means within a row with different letters significantly differ (P ≤ 0.01)*.

L* *, Lightness*;

a* *, redness*;

b* *, yellowness*;

H* *, hue angle*;

C* *chroma*.

Previous works have revealed lower lightness (< 40) and yellowness values (5.93), and a relatively higher redness value (13.18) in Spanish Brown Swiss, Pirenaica and Holstein Friesian breeds (54, 55). An appropriate chroma value and a higher hue value were also observed in comparison with data reported in the literature (17.96 and 28.74, respectively) (54). Both groups showed a chroma value approaching 18. Overall, the color parameters were significantly affected by the storage time (*P* < 0.01), but not by dietary treatments, as previously demonstrated in other studies using linseed as an ingredient in the concentrate feed during the finishing period (41, 42, 55). The beef color did not change at 24 h, after which the redness, yellowness and chroma values gradually decreased, as also reported by Albertì et al. (18). Lightness values decreased after 2 days of storage and, subsequently, remained stable (Table 7). Similarly, the redness (a^*^) and yellowness (b^*^) significantly decreased after 2 days of storage. Hue values increased over the storage time, whereas the chroma values had an opposite trend. Starting from the second day of shelf-life, in both groups the chroma value decreased to a value below 18. Beef with a chroma value of below 18 is not considered acceptable by consumers (56).

The lack of effect of linseed supplementation on the temporal pattern of beef color suggested that in minced beef, changes in the FA composition of lipids were not associated with the higher risk of oxidation of the heme pigments (18). In both treatments, changes in color stability during the storage period were probably due to the oxidative stress associated with the grinding process. Liu et al. (57) proposed enriching the diet of beef cattle with vitamin E at a level of 1.2 μg/g of muscle in order to increase the color stability during storage.

#### Lipolysis

The level of FFA in minced beef increased significantly after 6 days in storage at 4°C in both groups, irrespective of the dietary treatment (from 0.43± % of total lipids in fresh beef to 0.75%± and 1.71± in beef stored for 2 days and 4 days, respectively; *P* = 0.05). FFAs represent the product of the first step in the lipid peroxidation of raw meat, because of the lipolysis of triacylglycerols, and phospholipids due to the action of various lipolytic enzymes, such as ATGL (adipose triglycerides lipase), HSL (hormone sensitive lipase), MGL (monoacylglycerol lipase), and phospholipases A1 and A2 (58). These enzymes remain active even after the animal slaughtered (35) and thus, the amount of FFA represents a proxy of the first step of lipid peroxidation. The higher PUFA n-3 level in the L diet group did not affect the lipolysis process of triacylglycerols during the shelf-life, thus demonstrating that the hydrolysis of acylglycerols was not influenced by the nature of the fatty acids.

#### TBARS

**Figure 1:**
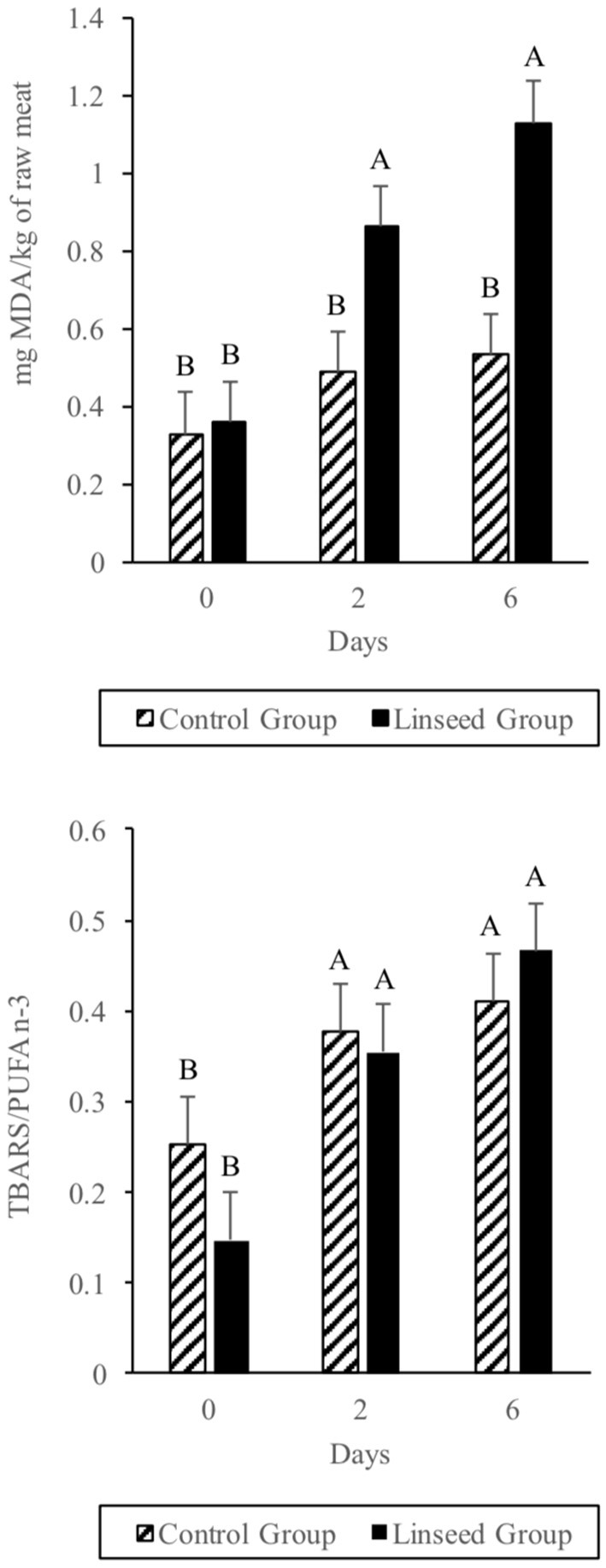
Effect of Linseed supplementation and storage time on TBARS level (up) and TBARS/PUFA (down). A-B Means with different letters differ in terms of interaction between diet and storing time factors (*P* ≤ 0.01).

Regarding secondary oxidation products, no differences (*p* > 0.05) between the two groups in terms of TBARS values were observed at 0 days (Figure 1 up). After 2 days of storage, the TBARS content increased significantly in the L diet group (*p* ≤ 0.05) and at the end of the storage period, the TBARS content was twice (1.2 μg MDA/g meat; *p* ≤ 0.05) that of the beef samples from animals fed the C diet (0.6 μg MDA/g meat). This value was much lower than the 3 mg MDA/Kg meat which is considered as the rancidity perception threshold, although many consumers can detect rancidity when MDA reaches 2 mg MDA/Kg meat (59). The higher TBARS value of the L diet beef was due to the higher content of α-linolenic acid in the intramuscular fat (60). In fact, considering the TBARS:PUFA n-3 ratio, there were no differences between treatments (Figure 1 down). This can be explained by the fact that MDA comes from the oxidation of fatty acids with more than two double bonds through the breakdown of cyclic peroxides (61).

Our results in minced beef were much lower than those reported in other similar studies (62). The period of grazing before the finishing phase probably enhanced the level of antioxidant compounds in both groups, with a subsequent reduction in beef oxidation that began immediately after the start of retail display. On the other hand, the TBARS values found in this study were much higher than those found in other experiments based on the addition of antioxidant products (63). Our data confirmed the important role of antioxidant substances during the finishing period in order to reduce beef oxidation during storage.

#### Cholesterol Oxidation Products

The total cholesterol content in the beef samples was not affected by dietary treatments, with an average concentration of 34 ± 0.76 mg/100 g of beef (Table 8). Since a high intake of COPs is related to an increased risk of several diseases in humans (64), there has been increasing interest in the analysis of COPs in animal-derived food. Five COPs were identified: 7β-hydroxycholesterol (7β-HC), 5-6 α-epoxycholesterol (5-6 α-EC), 5-6 β-epoxycholesterol (5-6 β-EC), 7-ketocholesterol (7-KC), and cholestantriol (triol). The most abundant COP in the beef samples was 7-KC (>50% of total COPs, Table 8), in line with previous works on fresh and processed pork and lamb meat (35).

**Table 8:** Effect of Linseed supplementation and storage time on vitamins and carotenoids (μg/Kg of raw meat), cholesterol (mg/100 g of raw meat), and COPs (μg/100 g of raw meat) in beef samples.

	**Control**			**Linseed**			**SEM^a^**	***P*****-value**		
	**T0**	**T2**	**T6**	**T0**	**T2**	**T6**		**D^b^**	**S^c^**	**DxS**
Cholesterol	32.91	31.97	31.07	32.09	31.87	31.95	0.77	0.77	< 0.01	0.50
7β-HC	24.06	14.35	14.23	18.18	12.70	22.95	3.28	0.87	0.07	0.08
5-6 β-EC	8.66^B^	47.07^A^	36.75^A^	19.51^B^	39.54^A^	56.20^A^	8.31	0.17	< 0.01	0.33
5-6 α-EC	21.84^a^	9.23^b^	16.75^b^	22.17^a^	10.60^b^	15.95^b^	4.37	0.94	0.02	0.96
Triol	0.00^B^	7.99^B^	8.74^B^	0.00^B^	6.32^B^	25.24^A^	4.45	< 0.01	< 0.01	0.05
7-KC	56.11^b^	37.65^b^	48.42^b^	63.38^b^	49.15^b^	93.16^a^	12.98	0.05	0.12	0.30
COPs total	109.90^b^	116.28^b^	124.90^b^	123.68^b^	118.58^b^	213.49^a^	20.62	< 0.01	0.02	0.05
COPs/chol.	0.32^B^	0.37^B^	0.39^B^	0.36^B^	0.37^B^	0.66^A^	0.06	0.05	< 0.01	0.05
Retinol	28.3^A^	17.9^B^	7.7^B^	38.7^A^	11.2^B^	1.2^B^	8.5	0.47	< 0.01	0.08
Lutein	7.7^A^	2.3^B^	1.0^B^	4.2^A^	2.6^B^	1.2^B^	0.8	0.21	< 0.01	0.07
β-caroten	32.1^A^	3.3^B^	1.9^B^	20.5^A^	5.3^B^	1.1^B^	6.6	0.27	0.02	0.21
α-tocopherol	50.8	23.3	8.2	58.9	40.3	23.4	5.2	0.13	< 0.01	0.46
γ-tocopherol	41.8	12.9	4.1	44.2	20.1	11.3	4.5	0.13	< 0.01	0.83
δ-tocopherol	6.4	2.3	1.8	5.8	2.7	2.1	1.5	0.32	< 0.01	0.18
?-tocopherol	100.2	38.9	14.1	108.9	62.8	40.1	10.0	0.12	< 0.01	0.48

a *Standard Error of Mean*.

b *Diet effect*.

c *Storage time effect. a,b, means within a row with different letters differ (P ≤ 0.05) A,B, means within a row with different letters differ (P ≤ 0.01). 7β-HC = 7β-hydroxycholesterol; 5–6 β-EC = 5–6 β-hydroxycholesterol; 5–6 α-EC = 5–6 α-epoxycholesterol; 7-KC = 7 ketocholesterol*.

Total COPs were significantly affected by dietary treatments (*P* < 0.01) and were higher in beef from animals fed with linseed (117.03 vs. 151.91 mg/100 g of muscle). In fact, the total cholesterol content did not change during storage, and at the end of storage, the COPs:Cholesterol ratio was significantly higher in the L diet beef samples (Table 8). These results are consistent with Vincenti et al. (65) and Luciano et al. (45) who evaluated the content of COPs in raw bovine and lamb meats, respectively. According to Luciano et al. (45), the trend of cholesterol oxidation is similar to that of FA peroxidation. In fact, cholesterol and unsaturated FA have the same oxidation pathway, and the cholesterol oxidation may also be accelerated by the presence of some products of FA peroxidation. The relatively low energy bond cleavage [78 kcal/mol (66)] of C7 hydrogen of cholesterol molecules, facilitates the production of alkyl radicals in C7 by the interaction of cholesterol with radical substances produced during FA oxidation. Subsequently, cholesterol is affected by additional chemical reactions producing hydroperoxides, which are then reduced to hydroxides, such as 7β-HC, a primary oxidation product of cholesterol. Hydroperoxides and hydroxides give rise to secondary oxidation products, namely 7-KC, 5-6 α-EC 5-6 **β**-EC, and triol. Thus, the COP content could be considered as a better proxy of lipid oxidation than the TBARS value, which, conversely, is only representative of the oxidation of FA with more than two double bonds (59). Thus, it is not surprising that storage time only affected the COPs of beef from animals fed linseed, for which the total content of COPs at the end of the storage time was nearly double. With respect to the individual COPs, 7β-HC increased significantly during storage. Considering that 7β-HC is a primary product of cholesterol oxidation, it may be that the process was in an early state. 7-KC, which is produced both by the dehydration of 7-hydroperoxide cholesterol and dehydrogenation of 7 hydroxycholesterol, showed a very similar trend as total COPs. On the other hand, as mentioned above, 7-KC is the main COP in beef and is thus considered as the best proxy of cholesterol oxidation (67). Epoxy 5,6 α-, and β-epimers, come from the epoxidation of 7-hydroperoxide cholesterol (67). These compounds are quite stable under physiological conditions, however in the presence of water and acidic conditions, they break down the epoxy ring, giving rise to cholestan triol (68). Because the α-epimer is more reactive than the β-epimer (64), the former decreased during conservation while the latter did not change. Triol was under detection limits in most of the samples at day 0, and subsequently increased with the storage time (*P* = 0.002) in line with the diminution of 5-6 α-EC. (Table 8).

The results of individual COPs were consistent with data obtained by Luciano et al. (45) in lamb meat stored for 4 days. To the best of our knowledge, little information is available on what quantity of COPs are necessary to induce a detrimental effect on human health. The negative effects of COPs are well described in the literature; however, no threshold has been proposed for human health. The effect of COP on human health is related to several factors, such as endogenous selective metabolism, type of oxysterols, and the endogenous status of organism. Previous studies (above all in animal models) have demonstrated that dietary oxysterols can be intestinally absorbed and transported by chylomicrons. Estimates of the extent to which oxysterols are absorbed range from 6 to 93%, according to the dose, type of tissue model and vehicle used to administer the oxysterol (68). It has been hypothesized that some oxysterols may be preferentially absorbed and transported by chylomicrons (68). It is also possible that some oxysterols (e.g., cholesterol epoxides) are hydrolyzed in the gut and that the selective metabolism of some oxysterols may occur during absorption of dietary sterols by the intestinal epithelium (66). However, Lercker and Rodriguez-Estrada (69) suggest that a COP/cholesterol ratio higher than 0.5% has a detrimental effect on human health. In this study, only beef samples from the L diet group, after 6 days of shelf-life, showed a higher value than this threshold.

#### Carotenoids and Vitamins

The total content of tocopherols was not significantly affected by linseed supplementation and was comparable to values reported in previous studies on beef obtained by grain-fed animals (60). To the best of our knowledge, the relationship between linseed supplementation and tocopherol content in meat has rarely been investigated. However, Focant et al. (70) reported that linseed supplementation increased the content of Vitamin E in milk fat.

The main tocopherol was α-tocopherol, which accounted for more than 50% of the total tocopherols.

The content of all tocopherols significantly decreased during the storage period, suggesting a protective effect of tocopherols against lipid oxidation. However, as reported above, the content of the oxidation products of FA and cholesterol was higher for the L diet beef samples, suggesting that a higher content of antioxidant substances was needed to tackle the oxidation of PUFA enriched beef. Liu et al. (71) and Gatellier et al. (72) reported that optimal protection against lipid oxidation during shelf-life, may be achieved when the vitamin E content was higher than 300 μg/100 g of beef.

Dietary treatments did not affect the carotenoid and retinol content, which, in beef samples were similar to those reported by Rohrle et al. (19) in the beef of Charolais x Limousine crossbred heifers, fed a concentrate-based diet. This therefore suggests that the effect of grazing before the finishing period was completely lost, similar to the findings reported above for tocopherols. Storage time significantly affected the level of carotenoids and retinol in beef samples, with a drastic reduction after just 2 days of storage. However, some of the tocopherols, carotenoids, and retinol may perhaps have been consumed during the aging period of the carcass as reported in a previous study (73).

According to the literature, β-carotene and lutein contents are positively associated with adipose tissue yellowness (74). In the present study, this relationship was not evident, as the decrease in carotenoid content after 2 days of storage, was not related to a simultaneous reduction in b^*^ values (Table 8).

## Conclusion

Linseed supplementation in the finishing period of young Maremmana bulls, after a grazing period, increased the content of n−3 PUFA (mainly C18:3 n-3) in beef with a consequent reduction in the n-6:n-3 ratio. However, the overall amount of n-3 PUFA provided by the L diet beef was far from the optimal amount for the daily requirements in the human diet. Further research is needed to develop feeding strategies aimed at increasing the n-3 content of beef to optimal levels for human health.

However, increasing the PUFA content of intramuscular fat resulted in a higher susceptibility to lipid oxidation, as demonstrated by the higher levels of TBARS and COPs after 2 days of storage, in beef samples from animals fed the L diet.

In conclusion, the increase in PUFA n-3 content in beef should be associated with higher levels of antioxidants than those normally present in the intramuscular fat. This should be coupled with packaging techniques capable of tackling the higher susceptibility to lipid oxidation, in order to control oxidation during storage, and to increase the shelf-life of beef.

## Ethics Statement

All the experimental procedures used in this study, followed the EU guidelines for the care and use of animals in research (Italian official bulletin no. 61, 2014).

## Author Contributions

GC, AS, AB, and MM conceived and designed the experiments and wrote the manuscript. LC, FC, AC, EB, and LC-R performed the experiments. All authors contributed to the interpretation of the data, and read, revised, and approved the final manuscript.

### Conflict of Interest Statement

The authors declare that the research was conducted in the absence of any commercial or financial relationships that could be construed as a potential conflict of interest.
